# Assessment of the impact of multiple mild-steam decontaminations on the protection performance of disposable KN95 filtering facepiece respirators

**DOI:** 10.1016/j.infpip.2021.100136

**Published:** 2021-03-03

**Authors:** F. Zentgraf, P. Johe, H. Hoche, B. Göckel, S. Becker, M. Oechsner, A. Dreizler

**Affiliations:** a Technical University of Darmstadt, Mechanical Engineering, Reactive Flows and Diagnostics, Otto-Berndt-Straße 3, 64287, Darmstadt, Germany; b Technical University of Darmstadt, Mechanical Engineering, Center for Structural Materials, Grafenstraße 2, 64283, Darmstadt, Germany; c Alice-SterilGutVersorgung, Alice-Hospital, Dieburger Straße 31, 64287, Darmstadt, Germany; d Division of Pediatric Pulmonology, Children's Hospital, Dieburger Straße 31, 64287, Darmstadt, Germany

**Keywords:** Sterilization, Face mask, Respirator, Filtration efficiency, KN95, COVID-19

## Abstract

The COVID-19 pandemic caused tremendous supply bottlenecks of single-use filtering facepiece respirators (FFRs) leading to a growing need for a potential reuse. This study assesses the impact of multiple mild-steam decontaminations with 121 °C/2000 mbar/20 min on the protection performance of disposable FFRs. It focuses on FFRs of type KN95 that is recently dominating the markets, but its decontamination is not covered in the literature. It was found that up to ten cycles, only minor degradation in the filter efficiency, breathing resistance and none in the material structure is apparent, suggesting a potential for multiple decontamination cycles at almost unchanged protective properties of KN95 FFRs.

## Introduction

The persisting COVID-19 pandemic caused worldwide supply bottlenecks of medical protective equipment, including single-use filtering facepiece respirators (FFRs) [1]. While international supply chains were impeded by national economy lockdowns and the export trade for health protection equipment was dropping to serve local markets, the private and medical customer demand was rising globally. The tremendous shortages highly impede recommended respiratory precautions against COVID-19 for employees and patients in the public health sector [2]. Here, the equipment shortages cause highly increased risks of COVID-19. In addition, the medical care for patients with other infectious diseases is complicated by the lack of protective equipment [3]. In China [4] and Italy [5] significant numbers of healthcare workers have been infected. Asymptomatic hospital staff infected with SARS-CoV-2 lead to nosocomial infections [6] of previously uninfected patients. A spread of the infection among health care workers worsens medical care. Thus, there is a growing need for a potential reuse of FFRs while maintaining the protection properties. While the European type FFP2 (test standard EN 149:2001+A1:2009) was no longer available during the onset of the pandemic, the Chinese type KN95 (test standard GB2626-2006) became indispensable in fighting the recent equipment shortages on the German market in specific and more general also on the European market. While reviews on decontaminating FFRs are existing, like by Polkinghorne and Branley [7], the effect of sterilization on KN95 FFRs in particular is not covered in the literature, as recently mentioned by Cai and Floyd [8]. The major aim of this study is to investigate the influence of one to ten cycles of a mild-steam sterilization scheme on the protection performance of type KN95 disposable FFRs and to extend the knowledge on KN95 reuse potential by decontamination.

## Methods

### Preparation and decontamination of FFRs

Six different FFRs were investigated with their manufacturer (product name) listed below. Since van Wezel *et al.* [9] recently pointed out the possibility that non-CE-certified FFRs, such as some KN95, do not fulfil the quality criteria of the European test standards EN 149:2001+A1:2009, benchmarking to a CE-certified reference appeared reasonable for the present study. Thus, a FFP2 FFR supplied by 3M (Aura 1862+) served as reference for EN 149:2001+A1:2009. Five type KN95 FFRs from Dongguan HuaGang Communication Technology (HG Disposable Face Mask), Guangdong Zhizhen Biological Medicine (DR.MFYAN), Guangzhou Meisu Industrial (Oany) and Zhongshan Futaiynan Medical Equipment (Daily Protective Face Mask and an unnamed type) were considered. The KN95 FFRs were rendered anonymous with labelling mask A-E. In order to prepare for testing, each FFR was split symmetrically. One half was sterilized, the second half served as the non-sterilized reference. This was done to account for the potential systematic bias from variations in the manufacturing process. Steam sterilization in an autoclave at 121 °C (250 °F)/2000 mbar for a duration of 20 minutes was applied (Appendix A, Figure A.1), because it is a well-known standard procedure (sterility assurance level (SAL) of 10^−6^) with a broad availability in the existing infrastructure of hospitals. The recommendations of the German Society for Hospital Hygiene (Deutsche Gesellschaft für Krankenhaushygiene e.V., DGKH) were followed in determining the sterilization conditions [10], in which according to DIN EN 285, saturated steam at 121 °C for 15 minutes is considered sufficiently effective to achieve the required SAL. Czubryt *et al.* [11] recently applied autoclaving at 121 °C for 30 minutes to reuse type N95 FFRs and proved the feasibility of the scheme for application in a major urban hospital. As reported in a preprint by Kumar *et al.* [12] standard autoclaving at 121 °C for 15 minutes was capable in sterilizing different type N95 FFRs from SARS-CoV-2, such that no viable and recoverable virus was detectable after one sterilization cycle. Accordingly, the procedure used here (121 °C/2000 mbar/20 min) is assessed as appropriate for sterile processing of used FFRs. The scheme did not apparently degrade the filter material and fit (assessed qualitatively) of type KN95 FFRs but may occasionally degrade elastic bands and/or bonding of nose clip starting from five cycles on (depending on the specific FFR type). Sterilization at temperatures >121 °C was found to cause irreversible damage.

### Analysis of filter efficiency and breathing resistance

The filter efficiency was assessed using laser diagnostics in a customized optically accessible flow test bench for simulating aerosol laden human inhalation and exhalation processes through FFR samples (Appendix A, Figure A.2). The test aerosol consisted of an air flow with dispersed tungsten carbide particles. The particles (mean: 280 nm, median: 200 nm, standard deviation: 115 nm, 97% <1 μm) are well-selected to cover relevant sizes of individual virions and agglomerations causing airborne/droplet-spread infections. However, it is emphasized that the aerosol in this study is different from the test standard EN 149:2001+A1:2009. A 20 mm diameter filter active region was probed in the test bench. The air flow was controlled at 3.3 l/min. This flow rate is scaled down from the 95 l/min for testing according to EN 149:2001+A1:2009, as only a fraction of the FFR is used. The Mie-scattering of the aerosol in front of and behind the sample was captured by a scientific camera. The number of particles was determined computationally within a 3 x 10 mm region for 100 individual images each sample. The ratio FE in equation (1) defines the filter efficiency in percentage. Additionally, the pressure difference over the sample was recorded.

(1) $$\text{FE}=\left(1-\frac{\sum_{i=1}^{100}\text{Particles behind FFR in image}\;i}{\sum_{i=1}^{100}\text{Particles in front of FFR in image}\;i}\right)\times 100\%$$

For statistical analysis, the recommendations of Bellolio *et al.* [13] were used to classify the measured data and select the required hypothesis tests. As the data is non-Gaussian distributed, median and quartiles were used. The statistical significance was assessed using both Kruskal-Wallis (as distribution test) and Wilcoxon rank sum tests (to test for a median variation).

The algorithmic implementation of the particle identification and the consecutive statistical analysis were done using MATLAB R2017b (The MathWorks, Inc.).

### Analysis of material

The microstructures of each of the four or five fabric layers were examined using a scanning electron microscope (SEM) type Zeiss EVO 60 XVP. To avoid image artifacts due to charging of the fabric by the electron beam, the samples were sputtered with gold layer (a few nanometres thick) and the acceleration voltage was set to 3 kV. Pictures were taken at various magnifications, i.e., 25x, 100x, 1000x and 5000x, respectively. The analyses were performed before and after the sterilization procedures. The goal was to identify whether there was a change in surface microstructure and particle adhesion behaviour due to the sterilization procedures. Moreover, a material analysis was done by Fourier Transform Infrared Spectroscopy (FT-IR).

The implementation of the procedures described in this study were approved for contingency hospital operation by the institutional review board for occupational health of the Alice Hospital Darmstadt. This study follows the Standards for Quality Improvement Reporting Excellence (SQUIRE 2.0) reporting guideline of the EQUATOR Network. The investigations were conducted from April 2 to May 15, 2020.

## Results

**Figure 1:**
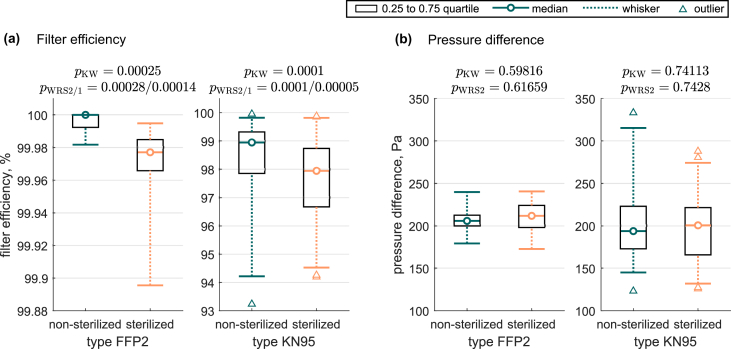
Influence of multiple sterilization on (a) filter efficiency and (b) pressure difference; all non-sterilized and corresponding sterilized samples summarized in classes (samples non-steri./steri.: 16/10 FFP2, 54/83 KN95). Dashed whiskers indicate the 0.025–0.975 quartile. Respective *P*-values are given on top of each plot.

**Figure 2:**
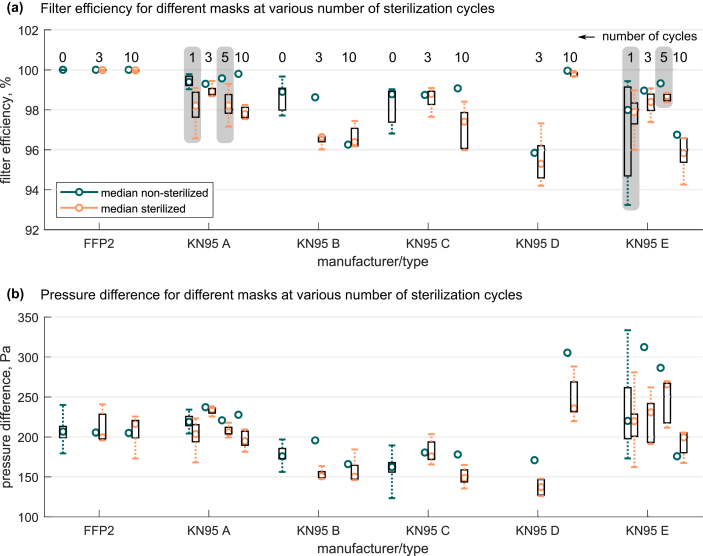
Statistical analysis of performance of individual manufacturers and impact of the number of sterilization cycles on (a) filter efficiency and (b) pressure difference. Boxes indicate the 0.25 to 0.75 quartiles and dashed whiskers the 0.025 to 0.975 quartiles. The numbers on top of each data column indicate number of sterilization cycles. Grey boxes in KN95 A and KN95 E are used for a visual separation to neighbouring specimen.

The influence of multiple sterilization cycles on filter efficiency and pressure difference is shown in Figure 1, summarizing all non-sterilized and corresponding sterilized samples in classes for both the FFP2 and KN95 type FFRs. Overall, medians for sterilized cases are about 0.02% (FFP2) and 1% (KN95) lower in the filter efficiency and approximately 6 Pa (FFP2) and 7 Pa (KN95) higher in the pressure difference, compared to the non-sterilized class. Including all outliers, the filter efficiency minimum is 99.90% (FFP2) and 93.23% (KN95) while the pressure difference maximum is 240 Pa (FFP2) and 333 Pa (KN95), respectively. For each corresponding pair (non-sterilized/sterilized), the Kruskal-Wallis test (null hypothesis H_0_: samples from the non-sterilized and sterilized class come from the same distribution; p_KW_) and two-sided Wilcoxon rank sum test (H_0_: median of non-sterilized and sterilized class are equal; p_WRS 2_) yield *P*-values of p_KW_=0.00025/p_WRS 2_=0.00028 (FFP2) and p_KW_=0.0001/p_WRS 2_=0.0001 (KN95) for the filter efficiency as well as p_KW_≈0.60/p_WRS 2_≈0.62 (FFP2) and p_KW_≈0.74/p_WRS 2_≈0.74 (KN95) for the pressure difference. A one-sided Wilcoxon rank sum test (H_0_: median of non-sterilized class < sterilized class; p_WRS 1_) for the filter efficiency features p_WRS 1_=0.00014 (FFP2) and p_WRS 1_=0.00005 (KN95). A comparison of the distribution of the filter efficiency of all exhalation samples to the corresponding inhalation samples resulted in p_KW_≈0.83 (both FFP2 and KN95). Figure 2 additionally breaks down the impact of individual manufacturers and the number of sterilisation cycles. In total 163 individual samples were extracted from 24 separate FFRs to compile the statistics on filter efficiency and pressure difference.

**Figure 3:**
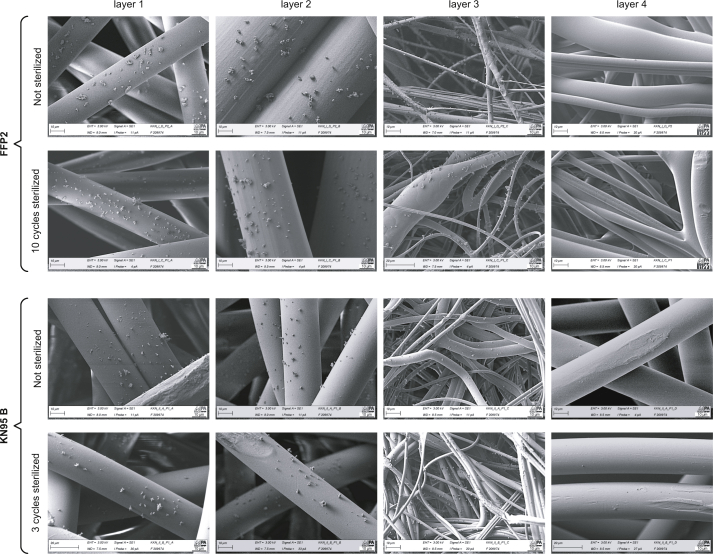
Layer-by-layer microstructure and particle adhesion of not sterilized/sterilized FFP2 and KN95 B samples. SEM images of the filter material. Individual scales are given in the left bottom corner of each sub-image.

An exemplary microstructural analysis by SEM for the FFP2 reference and KN95–B is summarized in Figure 3. Comparing the non-sterilized to the corresponding multiply times sterilized specimen layer-by-layer (1–4: environment-user), no visual evidence of a change or damage to the fabric structure as well as in the particle adhesion properties is found for both FFP2 and KN95–B. It is also observed that the filtering performance depends on the fabric structure, while filtering is achieved by the first three fabric layers only. The FT-IR revealed that all fabric layers were manufactured from polypropylene.

## Discussion

The results for the filter efficiency imply to reject the null hypothesis with a high statistical significance (*P* < 0.001). Thus, a difference between both the distributions (Kruskal-Wallis test) and medians (Wilcoxon rank sum test) of non-sterilized and sterilized samples is statistically highly significant. This finding is supported by the one-sided Wilcoxon rank sum test revealing consistently high significance for the alternative hypothesis (H_1_: median of non-sterilized class > sterilized class). Consequently, there is evidence for a deterioration in the filter efficiency due to sterilization up to ten cycles for both the FFP2 and KN95 type FFRs. However, despite statistical significance of differing filter efficiencies between non-sterilised and sterilised FFRs, it must be noted that for the median values only 0.02% deterioration for FFP2 and 1% for KN95 are observed. For practical relevance, this deterioration appears to be relatively low. For FFP2, 0.02% efficiency decrease appears even negligible considering the allowed particle transmissions up to 6% through the filter medium as specified in EN 149:2001+A1:2009 [14]. For the KN95 type FFRs, it should be as well considered that there is a significant scatter band observed for the samples of different manufacturers (see Figure 2). Comparing the non-sterilized KN95-A to E, 1.5% difference in the median is apparent (not shown here). Thus, the deterioration due to sterilization for KN95 FFRs is therefore in the same order of magnitude of the manufacturing-related variation and considered negligible in practical application. Comparing exhalation to inhalation probes, there is no evidence to reject the null hypothesis of equal distributions (Kruskal-Wallis test). This implies a maintained direction-independent protection after multiple sterilization. From the pressure differences, there is also no evidence to reject the null hypothesis of equal distributions (Kruskal-Wallis test) and equal medians (Wilcoxon rank sum test), due to the high *P*-values (>> 0.05). Here, the 6 Pa (FFP2) and 7 Pa (KN95) absolute increase from non-sterilized to sterilized FFRs also appears to be of minor relevance for practical in-field application. Despite the differences in the absolute values of the KN95 compared to the FFP2 FFRs as summarized in both Figure 1, Figure 2, this study is not attempting to assess the performance of KN95 masks according to the test standard EN 149:2001+A1:2009 for FFP2. This issue was recently addressed by van Wezel *et al.* [9], comparing non-CE-certified to certified respirators.

From the SEM images, it is observed that sterilization up to ten cycles does not indicate any damage of the surface of the fibres or their structure. It is concluded that the deterioration in filter efficiencies caused by sterilisation (0.02% for FFP2, 1% for KN95) are not visually evident in the SEM images. However, it remains unexplored if the applied sterilization procedure degrades the surface functionalization.

The study exhibits several limitations. The results only represent the behaviour of the six specific FFRs analysed here; the transferability to different FFRs might be limited. Furthermore, the method for measuring filter properties does not follow official certification standards, such as EN 149:2001+A1:2009. However, it assures a robust relative comparison between specimens. Additionally, only the filter efficiency of the filter material itself was tested, but no bypass on the edge of the FFR to the face. The fit of masks after decontamination was not quantified but rather assessed qualitatively.

## Conclusion

It was found that for up to ten sterilization cycles, a deterioration in the filter efficiency is statistically highly significant for both FFP2 and KN95 FFRs. However, the absolute values of deterioration (change in median: 0.02% FFP2, 1% KN95) are considered bearable for a practical in-field application. No significant degradation in terms of the breathing resistance and material structure is apparent for the investigated disposable FFRs. A decontamination for multiple uses by a 121 °C (250 °F)/2000 mbar/20 min mild steam sterilization scheme appears possible, while maintaining the protective properties relevant for practical in-field application. Depending on the KN95 FFR, secondary components like elastic bands or bonding of the nose clip did occasionally degrade starting from five cycles on, thus limiting the number of decontamination cycles.

## CRediT author statement

**Florian Zentgraf**: Conceptualization, Methodology, Software, Validation, Formal analysis, Investigation, Resources, Data Curation, Writing - Original Draft, Writing - Review & Editing, Visualization.

**Pascal Johe**: Conceptualization, Software, Formal analysis, Investigation, Resources, Data Curation.

**Holger Hoche**: Conceptualization, Methodology, Formal analysis, Investigation, Resources, Data Curation, Writing - Original Draft, Writing - Review & Editing, Visualization.

**Bernd Göckel**: Conceptualization, Methodology, Investigation, Resources, Writing - Original Draft.

**Sebastian Becker**: Conceptualization, Resources, Writing - Original Draft, Writing - Review & Editing.

**Matthias Oechsner**: Conceptualization, Methodology, Writing - Original Draft, Supervision, Project administration.

**Andreas Dreizler**: Conceptualization, Methodology, Writing Original Draft, Writing - Review & Editing, Supervision, Project administration.
