# Supplementary material for: Assessment of the impact of multiple mild-steam decontaminations on the protection performance of disposable KN95 filtering facepiece respirators
Source: Infect Prev Pract. 2021 Mar 3;3(2):100136. doi: 10.1016/j.infpip.2021.100136 (PMC8336038; doi:10.1016/j.infpip.2021.100136)
Supplement: Multimedia component 1 [file mmc1.docx]

**Appendix A – Supplementary data**

This appendix provides supplementary information for the Methods section by Figures A.1 and A.2.

**
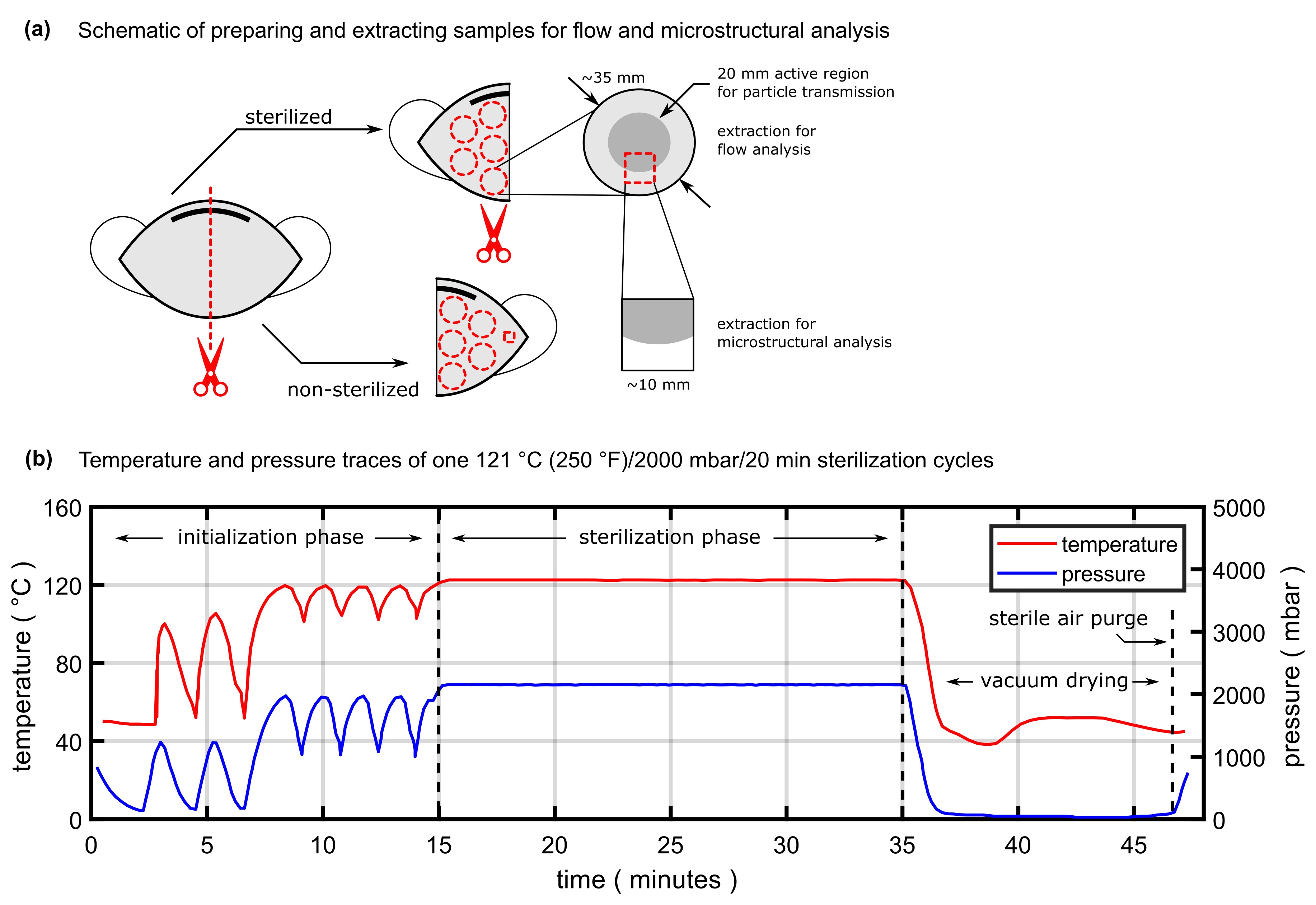
**

**Figure A.1.** (a) Preparation and extraction of FFR samples for analysis. (b) temperature and pressure time traces of one steam-sterilization cycle.

**
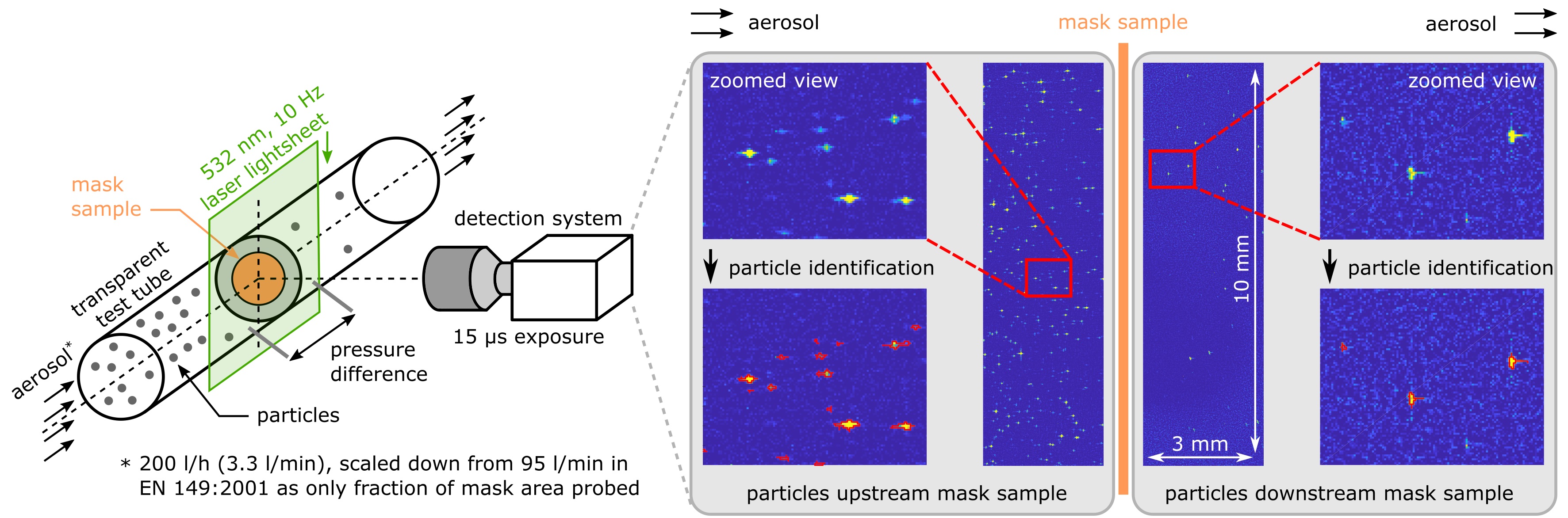
**

**Figure A.2.** Schematic of the flow-analysis test bench (left). Exemplary particle images up- and downstream the mask sample (right) with computationally identified particles framed red in the zoomed view.
